# The visual challenges of short-range navigation in teleost fish

**DOI:** 10.1242/jeb.250888

**Published:** 2026-02-11

**Authors:** Cait Newport, Theresa Burt de Perera

**Affiliations:** Department of Biology, University of Oxford, Oxford OX1 3EL, UK

**Keywords:** Spatial cognition, Vision, Turbidity, Object recognition

## Abstract

To understand how fish use vision to navigate, we must first understand what they see. This Review explores how visually guided navigation in teleost fishes is shaped by the structure of their visual systems, the cognitive processes that interpret sensory input and the dynamic environments they inhabit. With broad variation in habitat, ecology and visual capabilities, fish provide a powerful system for examining how sensory conditions influence navigation. We focus on short-range navigation and review core strategies – beaconing, pilotage, path integration and spatial mapping – alongside the visual and cognitive demands each entails. To assess which strategies are available to different species, we examine the visual processing pathway, from eye and retinal anatomy to behavioural evidence from cognition studies. These reveal that fish process visual information in a variety of ways to perform a diverse range of visual functions, including motion perception, object recognition and generalisation across viewpoint or lighting changes. We consider how sensory limitations and visual noise may constrain navigational accuracy, and how context or visual ability might shape which strategies are used. Environmental changes, such as turbidity, light pollution, or habitat degradation or shifts, can further degrade cue availability and reliability, affecting navigational performance. Understanding how visual information is received, processed and applied is therefore essential not only for interpreting observed behaviours, but also for predicting how fish may respond to changing environments. By linking sensory input with spatial behaviour, we propose a framework that integrates perception, cognition and movement, offering new insight into how diverse visual systems shape navigation across species.

## Teaser

**Summary:** This Review links visual perception to navigation by reviewing the visual demands of navigation strategies and the sensory capabilities of fish within the context of environmental challenges.

## Introduction

Navigation, broadly defined, is the process by which animals find their way from one location to another. It underpins essential behaviours such as finding food, avoiding predators, defending territories and locating mates (Healy, 1998). Thus, the strategies animals use to orient and travel through their environments, and the precision with which they do so, can profoundly affect their survival and reproductive success. For neuroethologists, studying how animals navigate helps us understand not just their movement, but also how they sense and respond to their surroundings, how their brains process information, and how they make decisions in the face of environmental uncertainty and complexity.

Teleost fishes are a particularly interesting group when considering navigation because of their phylogenetic diversity, wide variety of habitats and lifestyles, and the relative simplicity of their neural architecture compared with other vertebrates. These features make them useful for investigating how the environment, sensory systems and cognitive processing work together to guide navigation. Aquatic environments such as murky water, low light and changing visibility with depth or time of day also pose particular sensory challenges, which place additional demands on visual processing systems tasked with extracting reliable information. Finally, unlike surface-bound animals, fish movement is intrinsically three-dimensional. The addition of a vertical component increases the complexity of navigational tasks (Holbrook and Burt de Perera, 2009, 2011) and has major implications for an animal's cognitive load. These challenges have driven visual adaptations, making fishes ideal for studying how navigation is shaped by the sensory environment.

Despite growing evidence that fish can employ a variety of navigational strategies (Braithwaite and De Perera, 2006; Rodríguez et al., 2021), key questions remain unresolved. How is spatial information encoded in the brain? Do fish rely on associative learning or a more complex form of relational mapping to navigate (Guilford and de Perera, 2017)? Can they flexibly switch between navigational strategies depending on context or experience – and if so, what drives those switches? And how do differences in brain size, sensory capacity and ecological niche influence the types of navigational strategies different species favour? To advance our understanding of fish navigation, we argue that perception, cognition and the visual environment must be considered together. Perception refers to how fish detect and interpret visual cues in their surroundings. Cognition encompasses how this sensory information is stored, integrated and used to guide movement and decision-making. The visual environment includes the structural and optical properties of the habitat, which influence what information is available and how it can be used. Integrating these domains allows researchers not only to identify the mechanisms underlying navigation, but also to explore how sensory limitations or environmental noise might affect the accuracy and selection of different strategies. It also highlights how environmental change can disrupt or reshape the visual cues fish rely on to move through their world.

In this Review, we adopt a sensory-informed perspective to explore visual navigation in teleost fishes. We begin by outlining the range of navigational strategies observed in fish and the open questions that remain about the mechanisms underlying these behaviours. Our focus is on short-range navigation, rather than long-distance migrations, which are likely to rely on different processes. We then examine the anatomy and function of the fish visual system, emphasizing the capabilities most relevant to spatial orientation. Finally, we consider how visually guided navigation may be shaped or disrupted by environmental change. We aim to clarify how visual information supports navigation, and how sensory and cognitive limitations may constrain strategy use. In doing so, we hope to build a more integrated framework for studying navigation: one that connects behaviour with sensory biology and opens new directions for research.

Glossary **Landmark**

A recognizable feature in the environment that provides spatial information relative to a goal.

**Beaconing**

Navigating toward a visible landmark located at or very near the goal.

**Pilotage**

Following a learned sequence of landmarks or visual cues to reach a goal.

**Orientation**

Determining and maintaining a direction or heading relative to the environment.

**Path integration**

Continuously updating position and direction relative to a starting point or goal using cues arising from self-motion.

**Visual compass**

A directional cue derived from the overall visual scene used to maintain a consistent heading.

**Spatial map**

An internal representation of the environment that encodes the spatial relationships between locations and cues.

**Egocentric cue**

A spatial cue that defines location or direction relative to the current position of the navigator (e.g. turn left).

**Allocentric cue**

A spatial cue that defines locations or directions based on fixed relationships between landmarks, independent of the navigator's position (e.g. turn at the rock).

## Short-range navigation in teleost fishes

One of the simplest ways an animal can reach a goal is to use a specific landmark (see Glossary), or beacon, to identify the position of the goal (Braithwaite and De Perera, 2006). Warburton (1990) was among the first to show that goldfish (*Carassius auratus*) use beacons, by training fish to follow a single landmark to find a hidden food reward. Similarly, juvenile Atlantic salmon (*Salmo salar*) were trained to follow a unique landmark to locate a food patch (Braithwaite et al., 1996). One of the main benefits of beaconing (see Glossary) is that it is simple and likely requires minimal cognitive effort. However, it is not very useful when no suitable landmark is available near the target location. In such cases, one possible solution is to use a landmark that is relatively close to the goal, but not close enough to act as a beacon and learn the position of the target relative to that landmark. Warburton (1990) demonstrated that goldfish can also adopt this strategy. Given a choice of two food patches, one with and one without a landmark, the fish learned to swim to the patch without the landmark. In a small test tank, rather than act as a beacon, the landmark seems to provide an indirect cue, which the fish used together with the wall of the tank to pinpoint the position of the goal. One of the biggest limitations of beaconing is that it cannot be used if an animal is displaced, for example if a competitor or a predator chases an individual out of its home range. For beaconing to be effective, the fish needs to remain within the perceptual range of the landmark. This may be a particular issue for fish trying to navigate in low-visibility conditions. Thus, although beacons can effectively identify locations over a short range, they are unlikely to be used in isolation.

Some fish appear to use strategies that are still relatively simple but effectively extend their perceptual reach. By linking a series of familiar landmarks, an animal can navigate towards a goal that lies beyond the range of direct perception. This strategy, known as ‘pilotage’ (see Glossary), allows individuals to move through complex environments by progressing from one known location to the next. There are several reef fish species that appear to use pilotage and lists of landmarks to aid their orientation (see Glossary). For instance, Reese (1989) described how butterflyfishes (family Chaetontidae) follow predictable paths as they repeatedly swim between feeding patches on a reef. This behaviour broke down when prominent coral features were displaced experimentally, suggesting that the fish followed a learned sequence of landmarks. Similarly, brown surgeon fish (*Acanthurus nigrofuscus*) have been shown to change direction in accordance with the position of displaced landmarks, suggesting that they also orient using list-like information, following from one landmark to the next in a particular sequence (Mazeroll and Montgomery, 1998). This relatively simple piloting mechanism is route-based and does not require information about metric properties (distances and directions), but it does require that fish have a way of representing order or sequence (i.e. remembering where different landmarks are within their list). This behaviour is not just limited to the visual system – blind Mexican cave fish (*Astyanax fasciatus*), were able to use their lateral line organ to learn a specific sequence of landmarks arranged in a ring-shaped tank (Burt de Perera, 2004). To orient efficiently, the cave fish could identify their current position using the list, and then remember which place comes next. Similarly, salmonid fishes that migrate to sea after their juvenile phase in freshwater, learn the unique olfactory features that identify their home river on their outward journey (Dittman and Quinn, 1996). Once the fish are mature and preparing for reproduction, they reverse the list of imprinted odour cues and follow this sequence to find their natal stream (Dittman and Quinn, 1996). Lists of landmarks can therefore provide a means of moving over large distances beyond the perceptual range of a beacon and still enable fish to accurately home to a specific place.

Path integration (see Glossary) is a navigation strategy that does not depend on fixed landmarks. Instead, individuals continuously update information about the distance and direction of their movements to track their position relative to a starting point. By keeping track of how far and in which direction they have travelled, animals can, in theory, return to their point of origin via the most direct route, even across unfamiliar terrain and provided no obstacles block the way. This ability has so far been demonstrated in only one fish species. In a displacement experiment with shell-dwelling cichlids (*Lamprologus ocellatus*), Sibeaux et al. (2024) first allowed fish to swim from a home shell along an L-shaped route to a reward site. After this initial experience, the experimenters then displaced individuals and allowed them to head home again. Several fish swam directly towards the location where the home shell would have been, had it not been removed, consistent with the use of path integration. Path integration requires animals to estimate both the distance they have travelled and the direction of travel, two pieces of information that can potentially be extracted from visual input.

Freely swimming goldfish (Sibeaux et al., 2022) and Picasso triggerfish *Rhinecanthus aculeatus* (Karlsson et al., 2022), have both been shown to estimate distance travelled using visual information. In both experiments, the fish estimated distance travelled by integrating optic flow cues, which are based on the speed of visual motion generated as they move through the environment. Interestingly, the underlying mechanism by which fish achieve this differs fundamentally from terrestrial animals. Humans and terrestrial invertebrates measure the total angular motion of visual features for odometry, a mechanism which does not vary with spatial frequency of their environment. Animals such as bees will therefore experience a faster rate of angular change when flying close to a visual feature than one that is further away, a mechanism that they use for flight control (Srinivasan et al., 1997). In contrast, the visual odometer used by *R. aculeatus* is strongly dependent on the spatial frequency of their environment, and distance estimates are related to the number of contrast changes experienced on route. This means that their estimate of distance travelled does not depend on how far they are from the visual landmarks that generate the optic flow. Instead, they may estimate how far they have moved using a motion-detection system similar to the one responsible for the optomotor response — a separate system that both vertebrates and invertebrates use to stabilize their direction and gaze (Karlsson et al., 2022).

To measure direction within the path integration paradigm, fish may be able to use a compass (see Glossary) or an external reference that provides information about direction. Salmonids, for example, are thought to use a number of different compasses during long-distance migration, including the sun, geomagnetic fields and maybe even polarised light (Naisbett-Jones and Lohmann, 2022; Quinn, 2005). Even over short distances, other animals such as pigeons have been shown to use solar directional cues even within sight of their home loft (Armstrong et al., 2013). Despite this, no studies have tested whether fish use visual compasses for short-range path integration. Understanding which cues fish use to estimate direction will help us gauge the accuracy and robustness of path integration across species. It may also reveal that certain fishes, such as those living in deep or low-light environments where visual compass cues are unavailable, must instead rely solely on non-visual cues such as magnetic fields.

The accuracy of path integration likely depends on the specific mechanisms used to estimate distance and direction, and errors are likely to accumulate with increasing distance. The visually guided estimation systems at least appear to be inherently imprecise. In Sibeaux et al. (2022), fish trained to swim 70 cm averaged 73±16.77 cm, indicating substantial variability in optic flow-based estimation. While path integration benefits from relying on self-generated motion cues that are generally robust to changes in visibility or terrain, it may lack the precision needed to pinpoint locations accurately over longer distances, particularly in aquatic environments where water flow may introduce considerable noise. This imprecision could be further amplified in environments with obstacles that require frequent changes in direction. In support of this idea, Sibeaux et al. (2024) observed that some cichlids appeared to adopt alternative strategies, suggesting that path integration may not function as a standalone solution for navigation.

An ability to learn more complex relationships between landmarks could imply the use of a spatial map (see Glossary): an internal representation of the environment that encodes the geometric relationships between external cues. Forming a spatial map would allow fish to cope with unexpected displacement and navigate flexibly despite some changes to features. Rodríguez et al. (1994) demonstrated that goldfish can solve a simple T-maze using egocentric cues (see Glossary) such as ‘turn left at the junction’, using body position as a reference. The same fish could also learn the maze using allocentric cues (see Glossary), reaching a specific goal location regardless of turn direction, presumably by relying on visual landmarks in the surrounding room. Intriguingly, this behaviour was robust even when the experimenters rotated the maze, suggesting that the goldfish may have formed a spatial map based on the geometric configuration of external landmarks. Although evidence suggests that maps are not used at a large spatial scale in coral reef fish larvae (Spiecker et al., 2023), there is some evidence that magnetic maps may guide juvenile Pacific salmon, *Oncorhynchus tshawytscha* (Putman et al., 2014) and juvenile eels, *Anguilla anguilla* (Naisbett-Jones et al., 2017), over long ranges within oceans. These maps could not have been based on learned associations as the tested individuals were naïve, implying that maps at this large spatial scale are inherited.

Rather than relying on a single strategy, it seems likely that learned short-range spatial maps would be constructed through the integration of multiple navigational mechanisms. Path integration may provide a coarse spatial scaffold, with landmarks embedded into the animal's internal representation of space. Likewise, the sequential use of landmarks – as seen in pilotage – may contribute to a more structured and relational representation, helping to define not only where features are, but how they are connected. These combined processes suggest that spatial mapping in fish, although still not fully understood, may be supported by a flexible and layered use of available cues. This inherent flexibility may also enable fish to switch between different strategies when there are perturbations in the environment, for example, as caused by visual sensory pollution. *Danionella cerebrum* switch from using proximal landmarks to distal cues when subsets of the proximal cues are occluded (Lee and Briggman, 2023). Such context-dependent navigation may allow fish to deal with the challenges of changing visual conditions in natural environments.

### Visual demands of short-range navigation strategies

Each of the described navigational strategies, from beaconing to spatial mapping, presupposes certain visual and cognitive capabilities. In its most basic form, beaconing requires a fish to recognize a specific, learned landmark from multiple viewpoints (Braithwaite and De Perera, 2006; Warburton, 1990). Pilotage or following chains of landmarks requires the recognition of more than one object. In laboratory settings, where lighting and angles are stable, and the object features are simple (e.g. sphere, cube), this task may be relatively easy. In natural environments, however, where complex objects may appear different depending on water clarity, angle of approach, occlusion or background clutter, the visual system must support some form of object constancy to enable robust beacon recognition. Beyond recognition, the fish must also be able to search the visual scene to locate the relevant beacon among distractors. Available visual cues – shape, contrast, colour, texture – depend on visual acuity, spectral sensitivity and environmental conditions, all of which can influence which features are detectable and how reliably they can be identified during navigation under dynamic natural conditions.

Path integration requires the estimation of both the navigator's distance travelled and the direction of movement – tasks that can, but do not necessarily, rely on visual cues (Sibeaux et al., 2024). As previously discussed, distance can be inferred from visual motion cues such as optic flow, whereas direction may be guided by compass information derived from the sun or patterns of polarized light. Optic flow requires sensitivity to spatial frequency and contrast changes, and its reliability may be reduced in low-contrast or sparse versus cluttered environments. Celestial and polarization cues can provide reliable directional information, but their availability is limited to certain times of day or depths. Thus, the extent to which fish rely on vision for path integration probably varies with environmental context and species-specific visual capabilities.

Perhaps the most cognitively demanding strategy is the formation of a spatial map, as it requires the integration of multiple types of visual input. At a minimum, this includes the consistent recognition of individual landmarks from different viewpoints, the estimation of spatial relationships between landmarks and potentially the estimation of the navigator's distance to those landmarks using depth cues. These tasks could, in theory, rely entirely on vision. As with beaconing and pilotage, spatial mapping would also require the fish to actively search the environment, detect relevant features and recognize known objects within a potentially cluttered or visually noisy scene. Mapping, however, additionally demands that these visually derived cues be integrated into a coherent, allocentric representation of space, which may need to remain stable across different viewing conditions, such as changes in water clarity, lighting or the fish's orientation. In this sense, spatial mapping may place unique demands on the visual system not only for recognizing objects, but also for encoding spatial structure in a way that supports flexible and efficient navigation.

To fully understand navigation in fish (and indeed in any animal), it is essential to ground this investigation in the full sensory realities of the animals themselves: from how visual information is received by the eye to how it is processed by the brain. There is no use hypothesising that fish use polarised light as an orientation cue if their visual system doesn't allow them to see polarised light. Likewise, if a species lacks the spatial resolution to distinguish fine visual features, or if its environment is too dim or turbid for certain cues to be reliable, then some strategies may be functionally inaccessible. By carefully considering these sensory differences, we can better interpret observed behaviours and begin to disentangle which navigational strategies are supported by the visual systems of different species. We must also bear in mind that these visual systems have evolved in response to the specific conditions of each species' environment, which may differ in the availability of useful landmarks or in baseline visibility conditions.

## The visual capabilities of fish

Understanding how fish use vision to navigate first requires considering what their visual system allows them to perceive. Although broadly conserved, fish visual systems reflect adaptation to diverse ecological niches. Some species are adapted to dim, murky environments, whereas others rely on high-acuity vision in brightly lit, complex habitats. These differences influence what kinds of visual information fish can detect and use during navigation. In this section, we highlight key aspects of visual function that are most relevant to navigation, taking a broad perspective rather than focusing on individual species or ecological niches.

### Visual processing in the eyes

Broadly speaking, the anatomy of the visual system is consistent across vertebrates. The visual pathway starts as light enters the eye through the cornea, and the lens focuses light onto the retina. Teleost eyes, however, have structural differences from those of terrestrial vertebrates which lead to differences in how incoming light is focused. For example, the cornea of terrestrial animals has a significantly different refractive index than the surrounding air (Sivak, 1990). This difference in refractive index plays a large part in allowing incoming light to converge onto the retina, and ocular muscles and ligaments can alter the shape of primarily the cornea, but also the lens, to adjust focus (Ott, 2006). In teleosts, the difference in refractive index between the cornea and the surrounding water is negligible, and the main function of the cornea appears to be protecting the inner eye from external threats such as debris or ocular pressure (Collin and Collin, 2001; Jagger, 1992).

The lens is also different between aquatic teleosts and terrestrial vertebrates. While most terrestrial lenses are ellipsoid or biconvex (Ott, 2006), teleosts have a rigid, spherical lens (Jagger, 1992). These lenses have a smaller depth of field than human eyes owing to a short focal length to pupil diameter (Jagger, 1992; Kröger et al., 1999). Because of the optics associated with their shape, spherical lenses, such as those made of glass, can have limitations in optical performance, including reduced resolution at the edges and chromatic aberrations. However, fish eyes have evolved to compensate for these effects, and like terrestrial vertebrates, their lenses can focus a clear image onto the retina (Cronin et al., 2014). In addition, although teleosts cannot change lens shape to adjust focus, they can achieve accommodation by physically moving the lens within the eye (Fernald, 1988; Sivak, 1975). There are inherent limitations to this mechanism, however, as the extent of lens movement, and thus the range of visual accommodation, is constrained by the overall size of the eye (e.g. cornea-to-lens ratio) and these constraints differ among species (Fernald, 1988; Fernald and Wright, 1985; Sivak, 1975). Despite these anatomical differences, at this stage of the visual pathway the primary limitation on visual resolution, and thus feature detection, is the absolute size of the eye and lens.

The retina is responsible for converting the focused light into neural signals that can be processed by the brain. Like other vertebrates, the teleost retina has two types of photoreceptors – rods and cones – that detect light and play a role in visual perception. Rods are specialised for low-light (scotopic) conditions and support low-resolution, monochromatic vision, contributing to visual functions such as motion detection (Cronin et al., 2014; Schaerer and Neumeyer, 1996; Yokoyama, 2008). In fishes that inhabit predominantly scotopic environments, rod cell organisation can be adapted by increasing the number of rod layers to enhance the speed and sensitivity of visual responses (Fogg et al., 2023a). As a result, although many fish are capable of seeing in low-light conditions, their reliance on rod-based vision is likely to limit their ability to perceive fine details such as texture or colour, which largely depend on cone photoreceptors.

Cones operate in bright light (photopic) and are associated with higher acuity and colour vision. Each cone type is sensitive to a specific range of wavelengths. Humans have three cone types that are sensitive to short (420 nm), middle (534 nm) and long (564 nm) wavelengths, which respectively equate to our blue, green and red sensitivity (Bowmaker and Dartnall, 1980). In contrast, the number of spectrally distinct cone types in teleosts is species-specific and ranges from one to five. The spectral sensitivities of fishes are generally tuned to their ambient light environment (Bowmaker, 1995; Carleton et al., 2020; Yokoyama, 2008) and, as a result, some fish species can perceive a broader spectrum than humans, including ultraviolet wavelengths (<400 nm), while others may have more restricted colour vision (Losey et al., 1999). In addition to spectral tuning, cone organisation also influences visual capabilities. For example, the presence of double cones is thought to enable polarisation sensitivity (Cameron and Easter, 1993; Hawryshyn et al., 2003; Marshall and Cronin, 2011; Parkyn and Hawryshyn, 1993; Rowe et al., 1994). So far, only a few species, including northern anchovies (*Engraulis mordax*), have been shown to behaviourally respond to polarized light (Davitz and McKaye, 1978; Mussi et al., 2005; Novales Flamarique, 2017). In the anchovy, this capability led to a doubling in prey sighting distance (Novales Flamarique, 2019), indicating a potential evolutionary advantage for species that can exploit polarisation cues. Taken together, these features illustrate that many fish species not only perceive colour but, in some cases, can detect aspects of the visual world that are entirely beyond human capability. Given the diversity in cone types, spectral tuning and light detection strategies, it is essential to consider how species-specific visual systems shape their perception – even among species that share the same environment.

The organisation and density of rods and cones are also critical for determining the spatial resolution of the eye. In many species, regions of densely packed cones provide enhanced visual acuity. A fovea, for example, is a small pit of high-density cones that supports sharp central vision in humans (as well as other vertebrates) and is also found in some teleosts. Owing to this high cone density, the fovea is thought to play a key role in high-acuity tasks such as object identification. In teleosts without a distinct fovea, comparable regions of specialisations, such as horizontal streaks or temporal areas, can similarly increase acuity (Hofmann and Gebhardt, 2023). The position of these specialised regions is often aligned with ecological demands. For example, plankton-feeding species can have a forward-facing area centralis (AC), whereas benthic feeders tend to have one oriented downward to monitor the substrate (Collin et al., 2000; Collin and Pettigrew, 1988, 2008). These adaptations allow fish to direct their sharpest vision towards behaviourally relevant regions of their environment. A recent survey of 211 teleosts found 59 species that possessed a fovea, an additional 59 had an area centralis or horizontal streak, and 66 had regional specialisations (Hofmann and Gebhardt, 2023). While the sample is likely biased towards positive results and specific phylogenetic groups, these findings suggest that many teleosts possess some form of regional specialisation supporting high-acuity vision.

Having progressed through the optical components of the eye and the photoreceptor layer, we now reach the inner retina, where signals are processed and relayed by bipolar, amacrine and horizontal cells before reaching the retinal ganglion cells (RGCs). These RGCs serve as the final stage of retinal processing before visual information is transmitted to the brain (Masland, 2012). They play a central role in determining visual acuity, as each RGC effectively represents a ‘pixel’ of the visual field (Caves et al., 2023; Lee and Stevens, 2007; Pettigrew et al., 1988; Schellart and Rikkert, 1989). Owing to physical constraints on photoreceptor size, the angular density of RGCs sets an upper limit on the spatial resolution of the eye (Caves et al., 2023). RGCs can also act as feature detectors tuned to specific characteristics of the visual scene or object. For example, some RGCs are ON or OFF cells, responding to features such as size, movement direction, orientation, and colour (Abbas and Meyer, 2014; Aliper et al., 2021; Cronly-Dillon, 1964; Jacobson and Gaze, 1964; Kawasaki and Aoki, 1983; Maximova et al., 2021). Together, the density and functional diversity of retinal ganglion cells help define not only how sharply fish can see, but also what kinds of visual features they are capable of detecting and using to guide behaviour.

The average teleost eye has lower visual acuity compared with terrestrial visual specialists like birds of prey and humans (Caves et al., 2017). Visual acuity among teleosts, however, spans a wide range. For example, in a review of 81 marine species, the species with the highest measured acuity had vision approximately 45 times sharper than that of the lowest-performing species (Caves et al., 2017). Whether species have higher-acuity vision appears to be driven by habitat complexity rather than ambient light levels, turbidity or diet, probably because visually complex environments demand finer spatial resolution to detect and discriminate objects or features (Caves et al., 2017; Collin and Pettigrew, 1988, 2008). Depth also plays a key role: species living in deep, dim waters generally show a low acuity, whereas those inhabiting shallower depths (>600 m) often have a higher acuity, probably adapted for pattern recognition (Caves et al., 2023).

Although not strictly part of the visual processing pathway, it is important to consider the visual field and eye movements of fish, as these characteristics influence how much of the environment a fish can sample at any given time. Most fishes can move their eyes independently of body movement, and in many cases, these eye movements are coordinated. In a survey of 142 fish species, Hofmann and Gebhardt (2023) found 110 species that exhibited conjugate eye movements, in which both eyes move in coordination. Much of what we know about the function of eye movements comes from other vertebrate groups, often with very different eye positions. We have learned that eye coordination can facilitate focusing on the frontal field where binocular overlap is possible, supporting depth perception and potentially enhancing tasks such as prey capture or obstacle avoidance. Coordinated eye movements can also be important for gaze stabilization during self-motion, helping to reduce motion blur (Angelaki and Hess, 2005) – a function that may be supported by the presence of saccadic eye movements in many fish (Mandecki and Domenici, 2015). However, not all fish species use conjugate eye movements. In the same survey, 32 species displayed independent eye movements, allowing them to monitor different regions of their visual field simultaneously (Hofmann and Gebhardt, 2023). While this may be advantageous for detecting threats or tracking multiple objects, it can limit binocular overlap and thus may reduce the capacity for stereoscopic depth perception. Alternative depth cues, such as motion parallax, could potentially compensate for this, but to date, there is no direct evidence that fish use motion parallax as a visual cue for depth. Combined with their spherical lenses, which typically produce a wide visual field of around 180 deg (Easter et al., 1977), fish eye movements allow for the sampling of high-resolution visual information across much of their surroundings, even during movement. Nonetheless, our understanding of how fish direct their gaze during different tasks, including navigation, remains limited.

### Visual processing in the brain

While much research has focused on the capabilities and limitations of the eye itself, understanding visual navigation also requires examining how sensory input is interpreted and integrated at the neural level. Foundational work has identified key components of the visual processing pathways in fish, including the role of the optic tectum (Baier and Scott, 2024; Northmore, 2011). However, much of our knowledge on fish visual processing comes from just two model species, goldfish and zebrafish (*Danio rerio*), and typically centres on isolated visual tasks such as edge detection, orientation or colour processing. Our understanding of the brain mechanisms that support spatial learning and navigation in fish remains limited. Experiments using goldfish have shown that, like mammals, fish possess neural cells that represent environmental boundaries, head direction, and swimming speed and velocity vectors (Cohen et al., 2023; Vinepinsky et al., 2020). However, scientists have yet to find any evidence of the grid and place cells used to encode space in the mammalian brain (Hafting et al., 2005; O'Keefe and Dostrovsky, 1971). In our own brains, much of the visual processing occurs in the visual cortex (Grill-Spector and Malach, 2004). However, fish lack a cortex entirely, and so processing must be fundamentally different. For example, in fish, feature extraction and object categorization occur in the early stages of the visual pathway – unlike in primates, where these processes happen later (Volotsky and Segev, 2025). Given these substantial differences in brain structure and evolutionary history, we must be cautious when drawing analogies or inferring mechanisms from better-studied species. Thus, rather than focusing narrowly on the neural substrates themselves, we turn our attention to behaviour. Ultimately, behaviour provides the most direct window into how fish use vision to navigate, reflecting the integrated outcome of sensory input and brain processing in ecologically relevant contexts.

To understand how fish use visual cues to navigate, we must consider not only what their eyes are capable of, but how specific visual abilities contribute to locating, identifying and moving between spatial targets. Among the many visual processes involved, three stand out as particularly relevant given our previous discussion of the basic requirements for visually guided navigation: motion perception, depth estimation and object recognition. Each of these functions supports navigation in different ways, although their relative importance probably varies across species and contexts.

We begin with motion perception. The presence of motion-sensitive cells in the retina corresponds with behavioural evidence showing that fish respond reliably to moving stimuli. Optomotor response are reflexive movements to stabilise position in response to visual motion. They have been demonstrated in species such as goldfish (Schaerer and Neumeyer, 1996), Malawi cichlids (Smith et al., 2012) and zebrafish (e.g. Easter and Nicola, 1996). Zebrafish, which naturally inhabit turbid, low-visibility environments, show behavioural responses that may reflect adaptations for extracting optic flow information under such conditions. For example, they preferentially move toward sources of optic flow where motion signals are stronger (Scholtyssek et al., 2014) and their optomotor responses are primarily driven by motion in the lower lateral visual field, which is the region most likely to provide reliable optic flow in their natural habitat (Alexander et al., 2022). We have already seen that fish can use motion cues such as optic flow to estimate distance travelled (Karlsson et al., 2022; Sibeaux et al., 2022). In other animals, optic flow cues can also be used to estimate the distance of the observer to external objects, a function that supports obstacle avoidance and spatial orientation during navigation. However, it remains unclear whether fish use optic flow in this way, or how motion information is integrated in the brain and its role in spatial cognition. Interestingly, some species have been shown to perceive motion illusions (Gori et al., 2014), suggesting a level of central processing beyond simple reflexes.

Depth estimation is another potentially important visual ability for navigation, although far less is known about its neural basis in fish. While behavioural evidence exists, the mechanisms remain unclear. For instance, archerfish (*Toxotes chatareus*) demonstrate remarkable depth perception when they shoot down aerial prey with jets of water; an impressive feat given the optical distortions caused by the air–water interface (Newport and Schuster, 2020; Reinel and Schuster, 2018a,b). However, it remains unknown whether this ability relies on binocular overlap, monocular depth cues, other visual mechanisms or some combination of these. Furthermore, while depth estimation for prey capture clearly requires the fish to accurately judge spatial relationships between itself and a target, its broader usefulness to navigation remains uncertain.

Object recognition is the ability to identify and distinguish individual three-dimensional objects based on their visual features, such as shape, size, colour and texture, regardless of changes in position, orientation or lighting. All species of fish tested thus far are capable of at least simple 2D pattern discrimination. Species like trout, archerfish, triggerfish, damselfish and zebrafish can associate food rewards with coloured shapes, demonstrating shape–background discrimination (Brunet et al., 2023; Cheney et al., 2013; Newport et al., 2013; Santacà et al., 2021; Siebeck et al., 2009). So long as the shape is large enough, this ability appears to be generalisable across species regardless of variations in fish size, visual sensitivity, ecology or habitat. For example, both zebrafish (Sison and Gerlai, 2010) and rock bass *Ambloplites rupestris* (Halbrend et al., 2006) learned to find a red card and white tile, respectively, despite significant differences in their visual acuity (zebrafish: 0.87 cycles deg^−1^; rock bass: 40 cycles deg^−1^ Caves et al., 2017).

For a smaller number of species, experiments have been conducted on their ability to learn more complex shapes and patterns. For example, Ambon damselfish can learn to discriminate intricate conspecific UV facial patterns (Siebeck et al., 2010). Archerfish can discriminate between images of naturalistic objects, such as ‘spider’ or ‘leaves’ (Volotsky et al., 2022), as well as unfamiliar object classes such as human faces (Newport et al., 2016). Beyond that, fish are also capable of recognising patterns and objects despite the changes in appearance that are typical of natural viewing conditions, such as those caused by changing lighting conditions or individual motion. For example, goldfish (Douglas et al., 1988; Frech et al., 2012) and archerfish (Schuster et al., 2004) can learn the absolute size of an object and reliably select the correct stimulus even when its apparent size changes with viewing distance. Goldfish (Dörr and Neumeyer, 1997, 2000) and guppies *Poecilia reticulata* (Intskirveli et al., 2002) demonstrate colour constancy, and can identify a learned coloured stimulus despite changes in illumination. Archerfish can also generalise learned objects to novel viewpoints (Newport et al., 2018; Volotsky et al., 2022), meaning they can recognise objects even when viewed from new orientations or when some features are occluded owing to rotation. Similarly, the redtail splitfin (*Xenotoca eiseni*) is capable of discriminating 2D shapes despite partial occlusion (Sovrano and Bisazza, 2008).

However, the fact that some fish can perform visual tasks under controlled laboratory conditions does not necessarily mean we can predict how they will perceive or use navigational landmarks in more complex, natural settings. For instance, although some species demonstrate size constancy, they can also be misled by relative size illusions, where the perceived size of an object is influenced by surrounding elements (Lucon-Xiccato et al., 2019; Santacà et al., 2022, 2020). Similarly, fish can fall for brightness illusions, where the apparent brightness of an object changes depending on its background (Simpson et al., 2016). In addition, even though many fish appear capable of recognising potential landmarks across changes in movement and viewing angle, this flexibility also has limits. For example, archerfish can recognise rotated human faces, but performance breaks down at extreme angles, such as a 90 deg profile view (Newport et al., 2018). Although we do not yet know whether this pattern holds for other object classes, it raises the possibility that fish may have to adopt behavioural strategies to cope with their perceptual limits.

## Visually guided navigation in a changing world

Fishes possess a diverse range of visual capabilities adapted to the environments they inhabit. However, these environments are not static, they change over time due to both natural processes and more recently, human activity. Here we identify three major ways in which environmental change could affect visual navigation. First, visibility conditions may change. This can occur for a variety of reasons, but one common cause is increased turbidity, which can result from algal blooms, sediment runoff or other environmental disturbances (Caves and Johnsen, 2021; Dutkiewicz et al., 2019). Increased turbidity can reduce the detectability of visual landmarks and motion cues, and degrade perception of fine detail by scattering light and attenuating high spatial frequencies (Caves et al., 2017; Wells, 1969). It can also change the colour underwater (Marshall et al., 2019). Second, the features available to fish may change. Such changes may arise from a range of factors, including rising temperatures, coral bleaching, storm damage, or human activity – all of which can alter or eliminate familiar landmarks, change their appearance or increase the distances between them. Third, changes in light availability can further complicate navigation. Although turbidity is one cause of reduced light, other factors also play a role. For instance, fish that cannot escape warming surface waters – such as those in lakes – may be forced into deeper zones where the visual environment is markedly different. These shifts can alter both the structures available and the spectral properties of light, affecting colour perception and overall visibility (Caves and Johnsen, 2021). Conversely, some species may be exposed to artificial light pollution, which increases brightness at unnatural times and may disrupt visual rhythms (Bassi et al., 2022). Collectively, these environmental changes may displace individuals into unfamiliar or degraded habitats, potentially undermining the efficiency or effectiveness of the navigational strategies they typically rely on. Traits such as site fidelity or reliance on flexible navigation strategies like spatial mapping may determine how well different species adapt to these disruptions (Caves and Johnsen, 2021).

While many changes occur through natural processes, human activity may also increase their frequency, intensity, or timing in ways that are novel for some species. For example, in some regions, turbidity regularly increases during rainy seasons due to natural sediment runoff, and fish may be adapted to such fluctuations. However, if runoff also includes human-driven nutrient input, such as from agriculture or sewage, it can lead to algal blooms that not only increase turbidity but also shift the spectral composition of underwater light (e.g. making it greener), potentially altering the detection and perception of colour signals. Other anthropogenic changes, such as artificial light or large-scale habitat modification, may also fall outside the range of natural variation experienced by many species. The extent to which these environmental changes impact navigation likely depends on whether a given species or population has evolved in environments where similar visual variation occurs naturally, and on the flexibility of their underlying visual and navigational strategies. Without a clear understanding of the specific visual mechanisms that support navigation in teleosts, we cannot confidently predict how such changes will affect their spatial behaviour.

So, how might fish adapt? One possibility is through visual plasticity. Within each photoreceptor, opsin proteins are responsible for detecting light (Bowmaker, 2008). Research shows that vision in reef fish can exhibit plasticity over developmental time, seasonal cycles and across depth gradients (Cortesi et al., 2016; Stieb et al., 2016; Tettamanti et al., 2019). Some species, such as convict surgeonfish (*Acanthurus triostegus*), exhibit rapid plasticity in cone opsin expression under dim light, with changes occurring over just a few days (Fogg et al., 2023b). Changes under bright light were limited, suggesting that this plasticity is primarily triggered by substantial and sustained shifts in lighting conditions. Similar shifts have been observed in Malawi cichlids (Nandamuri et al., 2017). However, opsin plasticity is not universal. Damselfish, for instance, show highly species-specific responses to light changes (Stieb et al., 2016).

**Fig. 1.**
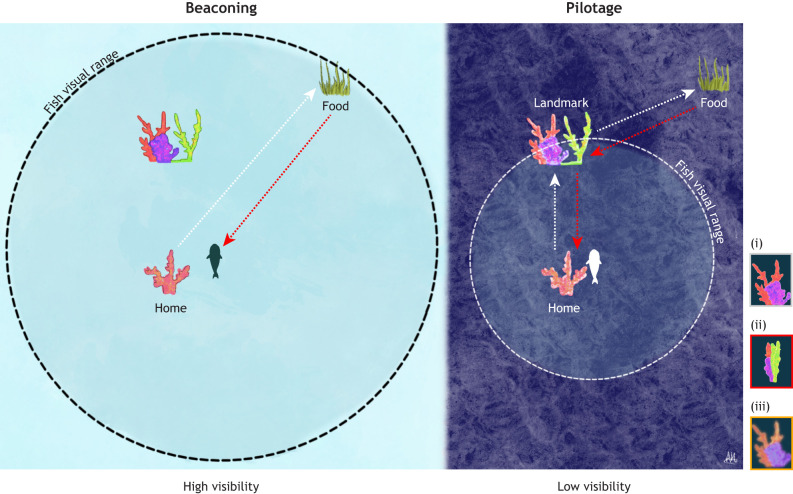
**Navigation strategies under different visibility conditions.** In clear water (high visibility), fish may rely on beaconing: when the target (e.g. a food source) is within visual range, they could swim directly towards it. On the way back, fish might use simple visual strategies, such as recognizing a familiar view of their home location and orienting towards it. White dashed lines indicate the outward journey, and red dashed lines the return journey. In low visibility, the target is no longer visible from the starting point. Direct movement based only on memory of its location is risky, as even small errors in heading can accumulate into large deviations and failure to reach the goal. Under these conditions, fish could instead adopt a pilotage strategy, following a sequence of nearby landmarks within their reduced visual range. However, pilotage may increase cognitive demands as more landscape features must be used as navigational cues and recognized under different conditions. For example: (i) a landmark as seen on the outward journey (white box); (ii) the same landmark appearing different on the return journey (red box); and (iii) the reduced clarity of landmarks under changing visibility. Original illustration by Alicia Hayden (www.aliciahayden.co.uk).

Behavioural and cognitive strategies may also help fish compensate for changes in visual conditions. For example, fish may begin to rely on landmarks that are closer together, more consistently visible, or simpler in structure (Fig. 1). Under this scenario, it is possible that closer or simpler landmarks may be more easily resolved under low visibility conditions, such as turbidity or low light, because they might be less affected by scattering, loss of contrast or reduced spatial resolution. While such adjustments could improve reliability under degraded conditions, they may also reduce navigational efficiency or increase the risk of error. In addition to these perceptual strategies, cognitive mechanisms may allow fish to cope with uncertainty. For example, Newport et al. (2024) found that Picasso triggerfish were able to complete a visual discrimination task by relying on learned positional likelihood when visual cues became indistinguishable, suggesting an ability to use prior probability information to guide decisions. Some species may also adopt navigational strategies, such as path integration, that are inherently less reliant on fine-scale visual detail and therefore more robust to sensory degradation. These behavioural and cognitive responses are particularly valuable because they can be deployed rapidly and flexibly, depending on the context and severity of sensory disruption. Unlike physiological adaptations, which may take several days to manifest, these strategies can offer immediate compensation for changing visual conditions.

Finally, it is possible that other sensory systems could contribute to navigation under low-visibility conditions. However, little is known about sensory integration in the context of fish navigation, and what evidence we do have largely comes from specialised taxa such as weakly electric fish – cases that may not generalise to most teleosts (Schumacher et al., 2016). The way sensory systems compensate for one another can vary: in some cases, different senses provide complementary information (e.g. one encoding spatial layout, another detecting motion); in others, multiple senses might encode the same object, with one being preferred under certain conditions (Schumacher et al., 2017). For instance, in electric fish, both vision and electrolocation can represent the same object, but which modality is relied upon depends on context (Schumacher et al., 2016). Whether such sensory system redundancy exists in typical teleost species remains unknown, and until it is better understood, it is difficult to assess how effectively non-visual senses can support navigation when visual information is unreliable. It is worth noting, however, that even if other sensory systems can compensate to some degree, they are unlikely to match the directional precision of visual cues, meaning that reductions in navigational accuracy and efficiency may still occur.

Ultimately, whereas plasticity, behavioural and cognitive adjustments, and other sensory modalities may offer some degree of compensation, visual cues provide spatial and directional information that is difficult, if not impossible, to replace. They allow fish to extract geometric structure, maintain orientation and identify meaningful features at a distance. As aquatic environments become more variable and less predictable, understanding how visual information is perceived, and integrated to produce behaviour will be critical to predicting how fish navigate and, by extension, how they survive.

## Summary

In this Review, we aim to connect research on vision with an ecologically significant behaviour. Specifically, we explore perception, cognition and movement through the lens of short-range navigation in fish. We examine how an animal's sensory experience shapes its encoding of space, and we integrate findings on how sensory disruptions influence both vision and navigation at short ranges in fish. The synthesis presented is intended to offer new insights into how diverse visual systems shape navigation across species.

## References

[JEB250888C1] Abbas, F. and Meyer, M. P. (2014). Fish vision: size selectivity in the zebrafish retinotectal pathway. *Curr. Biol.* 24, R1048-R1050. 10.1016/j.cub.2014.09.043 25517370

[JEB250888C2] Alexander, E., Cai, L. T., Fuchs, S., Hladnik, T. C., Zhang, Y., Subramanian, V., Guilbeault, N. C., Vijayakumar, C., Arunachalam, M., Juntti, S. A. et al. (2022). Optic flow in the natural habitats of zebrafish supports spatial biases in visual self-motion estimation. *Curr. Biol.* 32, 5008-5021.e8. 10.1016/j.cub.2022.10.009 36327979 PMC9729457

[JEB250888C3] Aliper, A. T., Damjanovic, I., Zaichikova, A. A., Maximova, E. M. and Maximov, P. V. (2021). Fine structure of the receptive fields of orientation-selective ganglion cells in the fish retina. *Neurosci. Behav. Physiol.* 51, 816-819. 10.1007/s11055-021-01138-7

[JEB250888C4] Angelaki, D. E. and Hess, B. J. M. (2005). Self-motion-induced eye movements: effects on visual acuity and navigation. *Nat. Rev. Neurosci.* 6, 966-976. 10.1038/nrn1804 16340956

[JEB250888C5] Armstrong, C., Wilkinson, H., Meade, J., Biro, D., Freeman, R. and Guilford, T. (2013). Homing pigeons respond to time-compensated solar cues even in sight of the loft. *PLoS ONE* 8, e63130. 10.1371/journal.pone.0063130 23717401 PMC3663752

[JEB250888C6] Baier, H. and Scott, E. K. (2024). The visual systems of zebrafish. *Annu. Rev. Neurosci.* 47, 255-276. 10.1146/annurev-neuro-111020-104854 38663429

[JEB250888C7] Bassi, A., Love, O. P., Cooke, S. J., Warriner, T. R., Harris, C. M. and Madliger, C. L. (2022). Effects of artificial light at night on fishes: a synthesis with future research priorities. *Fish Fish.* 23, 631-647. 10.1111/faf.12638

[JEB250888C8] Bowmaker, J. K. (1995). The visual pigments of fish. *Prog. Retin. Eye Res.* 15, 1-31. 10.1016/1350-9462(95)00001-1

[JEB250888C9] Bowmaker, J. K. (2008). Evolution of vertebrate visual pigments. *Vision Res.* 48, 2022-2041. 10.1016/j.visres.2008.03.025 18590925

[JEB250888C10] Bowmaker, J. K. and Dartnall, H. J. (1980). Visual pigments of rods and cones in a human retina. *J. Physiol.* 298, 501-511. 10.1113/jphysiol.1980.sp013097 7359434 PMC1279132

[JEB250888C11] Braithwaite, V. A. and De Perera, T. B. (2006). Short-range orientation in fish: how fish map space. *Mar. Freshw. Behav. Physiol.* 39, 37-47. 10.1080/10236240600562844

[JEB250888C12] Braithwaite, V. A., Armstrong, J. D., McAdam, H. M. and Huntingford, F. A. (1996). Can juvenile Atlantic salmon use multiple cue systems in spatial learning? *Anim. Behav.* 51, 1409-1415. 10.1006/anbe.1996.0144

[JEB250888C13] Brunet, V., Lafond, T., Kleiber, A., Lansade, L., Calandreau, L. and Colson, V. (2023). Environmental enrichment improves cognitive flexibility in rainbow trout in a visual discrimination task: first insights. *Front. Vet. Sci.* 10, 1184296. 10.3389/fvets.2023.1184296 37396987 PMC10313407

[JEB250888C14] Burt de Perera, T. (2004). Fish can encode order in their spatial map. *Proc. R. Soc. B* 271, 2131-2134. 10.1098/rspb.2004.2867 PMC1691836 15475332

[JEB250888C15] Cameron, D. A. and Easter, S. S. (1993). The cone photoreceptor mosaic of the green sunfish, *Lepomis cyanellus*. *Vis. Neurosci.* 10, 375-384. 10.1017/S095252380000376X 8485099

[JEB250888C16] Carleton, K. L., Escobar-Camacho, D., Stieb, S. M., Cortesi, F. and Marshall, N. J. (2020). Seeing the rainbow: mechanisms underlying spectral sensitivity in teleost fishes. *J. Exp. Biol.* 223, jeb193334. 10.1242/jeb.193334 32327561 PMC7188444

[JEB250888C17] Caves, E. M. and Johnsen, S. (2021). The sensory impacts of climate change: bathymetric shifts and visually mediated interactions in aquatic species. *Proc. R. Soc. B* 288, 20210396. 10.1098/rspb.2021.0396 PMC8059512 33878924

[JEB250888C18] Caves, E. M., Sutton, T. T. and Johnsen, S. (2017). Visual acuity in ray-finned fishes correlates with eye size and habitat. *J. Exp. Biol.* 220, 1586-1596. 10.1242/jeb.151183 28183870

[JEB250888C19] Caves, E. M., Sutton, T. T., Warrant, E. J. and Johnsen, S. (2023). Measures and models of visual acuity in epipelagic and mesopelagic teleosts and elasmobranchs. *J. Comp. Physiol. A* 209, 807-826. 10.1007/s00359-023-01661-7 PMC10465391 37572152

[JEB250888C20] Cheney, K. L., Newport, C., McClure, E. C. and Marshall, N. J. (2013). Colour vision and response bias in a coral reef fish. *J. Exp. Biol.* 216, 2967-2973. 10.1242/jeb.087932 23580729

[JEB250888C21] Cohen, L., Vinepinsky, E., Donchin, O. and Segev, R. (2023). Boundary vector cells in the goldfish central telencephalon encode spatial information. *PLoS Biol.* 21, e3001747. 10.1371/journal.pbio.3001747 37097992 PMC10128963

[JEB250888C22] Collin, S. P. and Collin, H. B. (2001). The fish cornea: adaptations for different aquatic environments. In *Sensory Biology of Jawed Fishes: New Insights* (ed. B. G. Kapoor, and T. J. Hara), pp. 57-96. Plymouth, UK: Science Publishers, Inc.

[JEB250888C23] Collin, S. P. and Pettigrew, J. D. (1988). Retinal topography in reef teleosts. I. Some species with well-developed areae but poorly-developed streaks. *Brain Behav. Evol.* 31, 269-282. 10.1159/000116594 3395836

[JEB250888C24] Collin, S. P. and Pettigrew, J. D. (2008). Retinal topography in reef teleosts: II. Some species with prominent horizontal streaks and high-density areae. *Brain Behav. Evol.* 31, 283-295. 10.1159/000116595 3395837

[JEB250888C25] Collin, S. P., Marshall, N. J., Shand, J., Chin, S. M., Harman, A. M. and Collin, S. P. (2000). The relationship between the position of the retinal area centralis and feeding behaviour in juvenile black bream *Acanthopagrus butcheri* (Sparidae: Teleostei). *Philos. Trans. R. Soc. Lond. Ser. B Biol. Sci.* 355, 1183-1186. 10.1098/rstb.2000.0663 11079394 PMC1692862

[JEB250888C26] Cortesi, F., Musilová, Z., Stieb, S. M., Hart, N. S., Siebeck, U. E., Cheney, K. L., Salzburger, W. and Marshall, N. J. (2016). From crypsis to mimicry: changes in colour and the configuration of the visual system during ontogenetic habitat transitions in a coral reef fish. *J. Exp. Biol.* 219, 2545-2558. 10.1242/jeb.139501 27307489

[JEB250888C27] Cronin, T. W., Johnsen, S. and Marshall, N. J. (2014). *Visual Ecology*. Princeton, NJ: Princeton University Press.

[JEB250888C28] Cronly-Dillon, J. R. (1964). Units sensitive to direction of movement in goldfish optic tectum. *Nature* 203, 214-215. 10.1038/203214a0 14207259

[JEB250888C29] Davitz, M. A. and McKaye, K. R. (1978). Discrimination between horizontally and vertically polarized light by the cichlid fish *Pseudotropheus macrophthalmus*. *Copeia* 1978, 333-334. 10.2307/1443573

[JEB250888C30] Dittman, A. and Quinn, T. (1996). Homing in Pacific salmon: mechanisms and ecological basis. *J. Exp. Biol.* 199, 83-91. 10.1242/jeb.199.1.83 9317381

[JEB250888C31] Dörr, S. and Neumeyer, C. (1997). Simultaneous color contrast in goldfish- a quantitative study. *Vision Res.* 37, 1581-1593. 10.1016/S0042-6989(96)00320-3 9231225

[JEB250888C32] Dörr, S. and Neumeyer, C. (2000). Color constancy in goldfish: the limits. *J. Comp. Physiol. A* 186, 885-896. 10.1007/s003590000141 11085641

[JEB250888C33] Douglas, R. H., Eva, J. and Guttridge, N. (1988). Size constancy in goldfish (*Carassius auratus*). *Behav. Brain Res.* 30, 37-42. 10.1016/0166-4328(88)90006-X 3166706

[JEB250888C34] Dutkiewicz, S., Hickman, A. E., Jahn, O., Henson, S., Beaulieu, C. and Monier, E. (2019). Ocean colour signature of climate change. *Nat. Commun.* 10, 578. 10.1038/s41467-019-08457-x 30718491 PMC6362115

[JEB250888C35] Easter, J. S. S. and Nicola, G. N. (1996). The development of vision in the zebrafish (*Danio rerio*). *Dev. Biol.* 180, 646-663. 10.1006/dbio.1996.0335 8954734

[JEB250888C36] Easter, S. S., Johns, P. R. and Baumann, L. R. (1977). Growth of the adult goldfish eye—I: optics. *Vision Res.* 17, 469-477. 10.1016/0042-6989(77)90041-4 878338

[JEB250888C37] Fernald, R. D. (1988). Aquatic adaptations in fish eyes. In *Sensory Biology of Aquatic Animals* (ed. J. Atema, R. R. Fay, A. N. Popper and W. N. Tavolga), pp. 435-466. New York, NY: Springer New York.

[JEB250888C38] Fernald, R. D. and Wright, S. E. (1985). Growth of the visual system in the African cichlid fish, *Haplochromis burtoni*: accommodation. *Vision Res.* 25, 163-170. 10.1016/0042-6989(85)90109-9 4013084

[JEB250888C39] Fogg, L. G., Chung, W.-S., Justin Marshall, N., Cortesi, F. and de Busserolles, F. (2023a). Multiple rod layers increase the speed and sensitivity of vision in nocturnal reef fishes. *Proc. R. Soc. B* 290, 20231749. 10.1098/rspb.2023.1749 PMC10688437 37989239

[JEB250888C40] Fogg, L. G., Cortesi, F., Gache, C., Lecchini, D., Marshall, N. J. and de Busserolles, F. (2023b). Developing and adult reef fish show rapid light-induced plasticity in their visual system. *Mol. Ecol.* 32, 167-181. 10.1111/mec.16744 36261875 PMC10099556

[JEB250888C41] Frech, B., Vogtsberger, M. and Neumeyer, C. (2012). Visual discrimination of objects differing in spatial depth by goldfish. *J. Comp. Physiol. A* 198, 53-60. 10.1007/s00359-011-0685-y 21960282

[JEB250888C42] Gori, S., Agrillo, C., Dadda, M. and Bisazza, A. (2014). Do fish perceive illusory motion? *Sci. Rep.* 4, 6443. 10.1038/srep06443 25246001 PMC4171700

[JEB250888C43] Grill-Spector, K. and Malach, R. (2004). The human visual cortex. *Annu. Rev. Neurosci.* 27, 649-677. 10.1146/annurev.neuro.27.070203.144220 15217346

[JEB250888C44] Guilford, T. and de Perera, T. B. (2017). An associative account of avian navigation. *J. Avian Biol.* 48, 191-195. 10.1111/jav.01355

[JEB250888C45] Hafting, T., Fyhn, M., Molden, S., Moser, M.-B. and Moser, E. I. (2005). Microstructure of a spatial map in the entorhinal cortex. *Nature* 436, 801-806. 10.1038/nature03721 15965463

[JEB250888C46] Halbrend, S. W., Davidson, H. S., M., H. J. and and Roosa, B. R. (2006). Rock bass learn to associate food with a visual cue and remember the association when food is absent. *J. Freshw. Ecol.* 21, 391-397. 10.1080/02705060.2006.9665015

[JEB250888C47] Hawryshyn, C. W., Moyer, H. D., Allison, W. T., Haimberger, T. J. and McFarland, W. N. (2003). Multidimensional polarization sensitivity in damselfishes. *J. Comp. Physiol. A* 189, 213-220. 10.1007/s00359-003-0392-4 12664097

[JEB250888C48] Healy, S. (1998). *Spatial Representation in Animals*. New York, NY, US: Oxford University Press.

[JEB250888C49] Hofmann, M. H. and Gebhardt, I. C. (2023). Evolution of the visual system in ray-finned fishes. *Vis. Neurosci.* 40, E005. 10.1017/S0952523823000020 38116689 PMC11016354

[JEB250888C50] Holbrook, R. I. and Burt de Perera, T. (2009). Separate encoding of vertical and horizontal components of space during orientation in fish. *Anim. Behav.* 78, 241-245. 10.1016/j.anbehav.2009.03.021

[JEB250888C51] Holbrook, R. I. and Burt de Perera, T. (2011). Three-dimensional spatial cognition: information in the vertical dimension overrides information from the horizontal. *Anim. Cogn.* 14, 613-619. 10.1007/s10071-011-0393-6 21452048

[JEB250888C52] Intskirveli, I. E., Roinishvili, M. O. and Kezeli, A. R. (2002). Experience-dependent color constancy in guppies (*Poecilia reticulata*). *Neural Plast.* 9, 205-216. 10.1155/NP.2002.205 12757371 PMC2565402

[JEB250888C53] Jacobson, M. and Gaze, R. M. (1964). Types of visual response from single units in the optic tectum and optic nerve of the goldfish. *Q. J. Exp. Physiol. Cogn. Med. Sci.* 49, 199-209. 10.1113/expphysiol.1964.sp001720 14141265

[JEB250888C54] Jagger, W. S. (1992). The optics of the spherical fish lens. *Vision Res.* 32, 1271-1284. 10.1016/0042-6989(92)90222-5 1455702

[JEB250888C55] Karlsson, C., Willis, J., Patel, M. and de Perera, T. B. (2022). Visual odometry of *Rhinecanthus aculeatus* depends on the visual density of the environment. *Commun. Biol.* 5, 1045. 10.1038/s42003-022-03925-5 36182985 PMC9526725

[JEB250888C56] Kawasaki, M. and Aoki, K. (1983). Visual responses recorded from the optic tectum of Japanese dace,*Tribolodon hakonensis*. *J. Comp. Physiol.* 152, 147-153. 10.1007/BF00611180

[JEB250888C57] Kröger, R. H., Campbell, M. C., Fernald, R. D. and Wagner, H. J. (1999). Multifocal lenses compensate for chromatic defocus in vertebrate eyes. *J. Comp. Physiol.* 184, 361-369. 10.1007/s003590050335 10377973

[JEB250888C58] Lee, T. J. and Briggman, K. L. (2023). Visually guided and context-dependent spatial navigation in the translucent fish *Danionella cerebrum*. *Curr. Biol.* 33, 5467-5477.e4. 10.1016/j.cub.2023.11.030 38070503

[JEB250888C59] Lee, S. and Stevens, C. F. (2007). General design principle for scalable neural circuits in a vertebrate retina. *Proc. Natl Acad. Sci. USA* 104, 12931-12935. 10.1073/pnas.0705469104 17646664 PMC1931479

[JEB250888C60] Losey, G. S., Cronin, T. W., Goldsmith, T. H., Hyde, D., Marshall, N. J. and McFarland, W. N. (1999). The UV visual world of fishes: a review. *J. Fish Biol.* 54, 921-943. 10.1111/j.1095-8649.1999.tb00848.x

[JEB250888C61] Lucon-Xiccato, T., Santacà, M., Miletto Petrazzini, M. E., Agrillo, C. and Dadda, M. (2019). Guppies, *Poecilia reticulata*, perceive a reversed Delboeuf illusion. *Anim. Cogn.* 22, 291-303. 10.1007/s10071-019-01237-6 30848385

[JEB250888C62] Mandecki, J. L. and Domenici, P. (2015). Eye movements are coordinated with pectoral fin beats during locomotion in a marine teleost fish. *J. Exp. Biol.* 218, 1122-1125. 10.1242/jeb.116756 25714565

[JEB250888C63] Marshall, J. and Cronin, T. W. (2011). Polarisation vision. *Curr. Biol.* 21, R101-R105. 10.1016/j.cub.2010.12.012 21300269

[JEB250888C64] Marshall, N. J., Cortesi, F., de Busserolles, F., Siebeck, U. E. and Cheney, K. L. (2019). Colours and colour vision in reef fishes: past, present and future research directions. *J. Fish Biol.* 95, 5-38. 10.1111/jfb.13849 30357835

[JEB250888C65] Masland, R. H. (2012). The neuronal organization of the retina. *Neuron* 76, 266-280. 10.1016/j.neuron.2012.10.002 23083731 PMC3714606

[JEB250888C66] Maximova, E. M., Aliper, A. T., Damjanović, I. Z., Zaichikova, A. A. and Maximov, P. V. (2021). Ganglion cells with sustained activity in the fish retina and their possible function in evaluation of visual scenes. *Neurosci. Behav. Physiol.* 51, 123-133. 10.1007/s11055-020-01047-1

[JEB250888C67] Mazeroll, A. I. and Montgomery, W. L. (1998). Daily migrations of a coral reef fish in the Red Sea (Gulf of Aqaba, Israel): initiation and orientation. *Copeia* 1998, 893-905. 10.2307/1447336

[JEB250888C68] Mussi, M., Haimberger, T. J. and Hawryshyn, C. W. (2005). Behavioural discrimination of polarized light in the damselfish Chromis viridis (family Pomacentridae). *J. Exp. Biol.* 208, 3037-3046. 10.1242/jeb.01750 16081602

[JEB250888C69] Naisbett-Jones, L. C. and Lohmann, K. J. (2022). Magnetoreception and magnetic navigation in fishes: a half century of discovery. *J. Comp. Physiol. A* 208, 19-40. 10.1007/s00359-021-01527-w 35031832

[JEB250888C70] Naisbett-Jones, L. C., Putman, N. F., Stephenson, J. F., Ladak, S. and Young, K. A. (2017). A magnetic map leads juvenile European Eels to the Gulf Stream. *Curr. Biol.* 27, 1236-1240. 10.1016/j.cub.2017.03.015 28416118

[JEB250888C71] Nandamuri, S. P., Yourick, M. R. and Carleton, K. L. (2017). Adult plasticity in African cichlids: rapid changes in opsin expression in response to environmental light differences. *Mol. Ecol.* 26, 6036-6052. 10.1111/mec.14357 28926160 PMC5690868

[JEB250888C72] Newport, C. and Schuster, S. (2020). Archerfish vision: visual challenges faced by a predator with a unique hunting technique. *Semin. Cell Dev. Biol.* 106, 53-60. 10.1016/j.semcdb.2020.05.017 32522409

[JEB250888C73] Newport, C., Sibeaux, A., Wallis, G., Wilkins, L. and Burt de Perera, T. (2024). An educated guess: how coral reef fish make decisions under uncertainty. *Anim. Behav.* 210, 245-254. 10.1016/j.anbehav.2024.02.016

[JEB250888C74] Newport, C., Wallis, G., Temple, S. E. and Siebeck, U. E. (2013). Complex, context-dependent decision strategies of archerfish, *Toxotes chatareus*. *Anim. Behav.* 86, 1265-1274. 10.1016/j.anbehav.2013.09.031

[JEB250888C75] Newport, C., Wallis, G., Reshitnyk, Y. and Siebeck, U. E. (2016). Discrimination of human faces by archerfish (*Toxotes chatareus*). *Sci. Rep.* 6, 27523. 10.1038/srep27523 27272551 PMC4895153

[JEB250888C76] Newport, C., Wallis, G. and Siebeck, U. E. (2018). Object recognition in fish: accurate discrimination across novel views of an unfamiliar object category (human faces). *Anim. Behav.* 145, 39-49. 10.1016/j.anbehav.2018.09.002

[JEB250888C77] Northmore, D. P. M. (2011). Optic tectum. In *Encyclopedia of Fish Physiology: From Genome to Environment*, vol. 1 (ed. A. Farrell), pp. 131-142. Online: Elsevier.

[JEB250888C78] Novales Flamarique, I. (2017). A vertebrate retina with segregated colour and polarization sensitivity. *Proc. R. Soc. B* 284, 20170759. 10.1098/rspb.2017.0759 PMC5597823 28878058

[JEB250888C79] Novales Flamarique, I. (2019). Swimming behaviour tunes fish polarization vision to double prey sighting distance. *Sci. Rep.* 9, 944. 10.1038/s41598-018-37632-1 30700806 PMC6353921

[JEB250888C80] O'Keefe, J. and Dostrovsky, J. (1971). The hippocampus as a spatial map. Preliminary evidence from unit activity in the freely-moving rat. *Brain Res.* 34, 171-175. 10.1016/0006-8993(71)90358-1 5124915

[JEB250888C81] Ott, M. (2006). Visual accommodation in vertebrates: mechanisms, physiological response and stimuli. *J. Comp. Physiol. A* 192, 97-111. 10.1007/s00359-005-0049-6 16172892

[JEB250888C82] Parkyn, D. C. and Hawryshyn, C. W. (1993). Polarized-light sensitivity in rainbow trout (Oncorhynchus mykiss): characterization from multi-unit responses in the optic nerve. *J. Comp. Physiol. A* 172, 493-500. 10.1007/BF00213531

[JEB250888C83] Pettigrew, J. D., Dreher, B., Hopkins, C. S., McCall, M. J. and Brown, M. (1988). Peak density and distribution of ganglion cells in the retinae of microchiropteran bats: implications for visual acuity. *Brain Behav. Evol.* 32, 39-56. 10.1159/000116531 3191381

[JEB250888C84] Putman, N. F., Scanlan, M. M., Billman, E. J., O'Neil, J. P., Couture, R. B., Quinn, T. P., Lohmann, K. J. and Noakes, D. L. G. (2014). An inherited magnetic map guides ocean navigation in juvenile pacific salmon. *Curr. Biol.* 24, 446-450. 10.1016/j.cub.2014.01.017 24508165

[JEB250888C85] Quinn, T. P. (2005). *The Behavior and Ecology of Pacific Salmon and Trout*. University of Washington Press.

[JEB250888C86] Reese, E. (1989). Orientation behavior of butterflyfishes (family Chaetodontidae) on coral reefs: spatial learning of route specific landmarks and cognitive maps. *Environ. Biol. Fishes* 25, 79-86. 10.1007/BF00002202

[JEB250888C87] Reinel, C. P. and Schuster, S. (2018a). Rapid depth perception in hunting archerfish. I. The predictive C-starts use an independent estimate of target height. *J. Exp. Biol.* 221, jeb177345. 10.1242/jeb.177345 29798847

[JEB250888C88] Reinel, C. P. and Schuster, S. (2018b). Rapid depth perception in hunting archerfish. II. An analysis of potential cues. *J. Exp. Biol.* 221, jeb177352. 10.1242/jeb.177352 29798848

[JEB250888C89] Rodríguez, F., Durán, E., Vargas, J. P., Torres, B. and Salas, C. (1994). Performance of goldfish trained in allocentric and geocentric maze procedures suggests the presence of a cognitive mapping system in fishes. *Anim. Learn. Behav.* 10, 108-114.

[JEB250888C90] Rodríguez, F., Quintero, B., Amores, L., Madrid, D., Salas-Peña, C. and Salas, C. (2021). Spatial cognition in teleost fish: strategies and mechanisms. *Animals* 11, 2271. 10.3390/ani11082271 34438729 PMC8388456

[JEB250888C91] Rowe, M. P., Engheta, N., Easter, S. S. and Pugh, E. N. (1994). Graded-index model of a fish double cone exhibits differential polarization sensitivity. *J. Opt. Soc. Am. A* 11, 55-70. 10.1364/JOSAA.11.000055 8106915

[JEB250888C92] Santacà, M., Lucon-Xiccato, T. and Agrillo, C. (2020). The Delboeuf illusion's bias in food choice of teleost fishes: an interspecific study. *Anim. Behav.* 164, 105-112. 10.1016/j.anbehav.2020.04.012

[JEB250888C93] Santacà, M., Dadda, M., Miletto Petrazzini, M. E. and Bisazza, A. (2021). Stimulus characteristics, learning bias and visual discrimination in zebrafish (*Danio rerio*). *Behav. Process.* 192, 104499. 10.1016/j.beproc.2021.104499 34499984

[JEB250888C94] Santacà, M., Bisazza, A. and Agrillo, C. (2022). Guppies (*Poecilia reticulata*) are deceived by visual illusions during obstacle negotiation. *Biol. Lett.* 18, 20210548. 10.1098/rsbl.2021.0548 35193367 PMC8864340

[JEB250888C95] Schaerer, S. and Neumeyer, C. (1996). Motion detection in goldfish investigated with the optomotor response is “color blind”. *Vision Res.* 36, 4025-4034. 10.1016/S0042-6989(96)00149-6 9068855

[JEB250888C96] Schellart, N. A. M. and Rikkert, W. E. I. (1989). Response features of visual units in the lower midbrain of the rainbow trout. *J. Exp. Biol.* 144, 357-375. 10.1242/jeb.144.1.357

[JEB250888C97] Scholtyssek, C., Dacke, M., Kröger, R. and Baird, E. (2014). Control of self-motion in dynamic fluids: fish do it differently from bees. *Biol. Lett.* 10, 20140279. 10.1098/rsbl.2014.0279 24872463 PMC4046384

[JEB250888C98] Schumacher, S., Burt de Perera, T., Thenert, J. and von der Emde, G. (2016). Cross-modal object recognition and dynamic weighting of sensory inputs in a fish. *Proc. Natl Acad. Sci. USA* 113, 7638-7643. 10.1073/pnas.1603120113 27313211 PMC4941484

[JEB250888C99] Schumacher, S., Burt de Perera, T. and von der Emde, G. (2017). Electrosensory capture during multisensory discrimination of nearby objects in the weakly electric fish *Gnathonemus petersii*. *Sci. Rep.* 7, 43665. 10.1038/srep43665 28257127 PMC5363222

[JEB250888C100] Schuster, S., Rossel, S., Schmidtmann, A., Jäger, I. and Poralla, J. (2004). Archer fish learn to compensate for complex optical distortions to determine the absolute size of their aerial prey. *Curr. Biol.* 14, 1565-1568. 10.1016/j.cub.2004.08.050 15341743

[JEB250888C101] Sibeaux, A., Karlsson, C., Newport, C. and Burt de Perera, T. (2022). Distance estimation in the goldfish (*Carassius auratus*). *Proc. R. Soc. B* 289, 20221220. 10.1098/rspb.2022.1220 PMC9554733 36476009

[JEB250888C102] Sibeaux, A., Newport, C., Green, J. P., Karlsson, C., Engelmann, J. and Burt de Perera, T. (2024). Taking a shortcut: what mechanisms do fish use? *Commun. Biol.* 7, 578. 10.1038/s42003-024-06179-5 38755224 PMC11099040

[JEB250888C103] Siebeck, U. E., Litherland, L. and Wallis, G. M. (2009). Shape learning and discrimination in reef fish. *J. Exp. Biol.* 212, 2113-2119. 10.1242/jeb.028936 19525438

[JEB250888C104] Siebeck, U. E., Parker, A. N., Sprenger, D., Mathger, L. M. and Wallis, G. (2010). A species of reef fish that uses ultraviolet patterns for covert face recognition. *Curr. Biol.* 20, 407-410. 10.1016/j.cub.2009.12.047 20188557

[JEB250888C105] Simpson, E. E., Marshall, N. J. and Cheney, K. L. (2016). Coral reef fish perceive lightness illusions. *Sci. Rep.* 6, 35335. 10.1038/srep35335 27748401 PMC5066220

[JEB250888C106] Sison, M. and Gerlai, R. (2010). Associative learning in zebrafish (*Danio rerio*) in the plus maze. *Behav. Brain Res.* 207, 99-104. 10.1016/j.bbr.2009.09.043 19800919 PMC2814798

[JEB250888C107] Sivak, J. G. (1975). Accommodative lens movements in fishes: movement along the pupil axis vs movement along the pupil plane. *Vision Res.* 15, 825-828. 10.1016/0042-6989(75)90261-8 1154663

[JEB250888C108] Sivak, J. G. (1990). Optical variability of the fish lens. In *The Visual System of Fish* (ed. R. Douglas and M. Djamgoz), pp. 63-80. Dordrecht: Springer Netherlands.

[JEB250888C109] Smith, A. R., Ma, K., Soares, D. and Carleton, K. L. (2012). Relative LWS cone opsin expression determines optomotor thresholds in Malawi cichlid fish. *Genes Brain Behav.* 11, 185-192. 10.1111/j.1601-183X.2011.00739.x 21992615

[JEB250888C110] Sovrano, V. and Bisazza, A. (2008). Recognition of partly occluded objects by fish. *Anim. Cogn.* 11, 161-166. 10.1007/s10071-007-0100-9 17636365

[JEB250888C111] Spiecker, L., Curdt, F., Bally, A., Janzen, N., Kraemer, P., Leberecht, B., Kingsford, M. J., Mouritsen, H., Winklhofer, M. and Gerlach, G. (2023). Coral reef fish larvae show no evidence for map-based navigation after physical displacement. *iScience* 26, 106950. 10.1016/j.isci.2023.106950 37378340 PMC10291465

[JEB250888C120] Srinivasan, M. V., Zhang, S. W. and Bidwell, N. J. (1997). Visually mediated odometry in honeybees. *J. Exp. Biol.* 200, 2513-2522. 10.1242/jeb.200.19.2513 9320443

[JEB250888C112] Stieb, S. M., Carleton, K. L., Cortesi, F., Marshall, N. J. and Salzburger, W. (2016). Depth-dependent plasticity in opsin gene expression varies between damselfish (Pomacentridae) species. *Mol. Ecol.* 25, 3645-3661. 10.1111/mec.13712 27262029

[JEB250888C113] Tettamanti, V., de Busserolles, F., Lecchini, D., Marshall, N. J. and Cortesi, F. (2019). Visual system development of the spotted unicornfish, *Naso brevirostris* (Acanthuridae). *J. Exp. Biol.* 222, jeb209916. 10.1242/jeb.209916 31776185

[JEB250888C114] Vinepinsky, E., Cohen, L., Perchik, S., Ben-Shahar, O., Donchin, O. and Segev, R. (2020). Representation of edges, head direction, and swimming kinematics in the brain of freely-navigating fish. *Sci. Rep.* 10, 14762. 10.1038/s41598-020-71217-1 32901058 PMC7479115

[JEB250888C115] Volotsky, S. and Segev, R. (2025). Object identity representation occurs early in the archerfish visual system. *Sci. Rep.* 15, 4102. 10.1038/s41598-025-88660-7 39900793 PMC11790826

[JEB250888C116] Volotsky, S., Ben-Shahar, O., Donchin, O. and Segev, R. (2022). Recognition of natural objects in the archerfish. *J. Exp. Biol.* 225, jeb243237. 10.1242/jeb.243237 35142811 PMC8918800

[JEB250888C117] Warburton, K. (1990). The use of local landmarks by foraging goldfish. *Anim. Behav.* 40, 500-505. 10.1016/S0003-3472(05)80530-5

[JEB250888C118] Wells, W. H. (1969). Loss of resolution in water as a result of multiple small-angle scattering. *J. Opt. Soc. Am.* 59, 686-691. 10.1364/JOSA.59.000686

[JEB250888C119] Yokoyama, S. (2008). Evolution of dim-light and color vision pigments. *Annu. Rev. Genomics Hum. Genet.* 9, 259-282. 10.1146/annurev.genom.9.081307.164228 18544031

